# Indestructible Wood

**Published:** 1854-04

**Authors:** 


INDESTRUCTIBLE WOOD.—Multitudes of human lives, and millions of treasure could be saved every year if our houses could be rendered fire-proof; there is no necessary obstacle to this, if, as is stated, the *Chihoe* tree in Mexico, becomes petrified in a very few years after it is cut, whether left in the air, or buried. It is a very fine grained wood, and is easily worked in a green state. If houses were built of it while in a green state, they would in a few years become as indestructible as if built wholly of iron or stone.

